# Transgenic Overexpression of LARGE Induces α-Dystroglycan Hyperglycosylation in Skeletal and Cardiac Muscle

**DOI:** 10.1371/journal.pone.0014434

**Published:** 2010-12-28

**Authors:** Martin Brockington, Silvia Torelli, Paul S. Sharp, Ke Liu, Sebahattin Cirak, Susan C. Brown, Dominic J. Wells, Francesco Muntoni

**Affiliations:** 1 Dubowitz Neuromuscular Centre, University College London (UCL) Institute of Child Health and Great Ormond Street Hospital, London, United Kingdom; 2 Department of Cellular and Molecular Neuroscience, Imperial College, London, United Kingdom; 3 Department of Veterinary Basic Science, Royal Veterinary College, London, United Kingdom; McMaster University, Canada

## Abstract

**Background:**

LARGE is one of seven putative or demonstrated glycosyltransferase enzymes defective in a common group of muscular dystrophies with reduced glycosylation of α-dystroglycan. Overexpression of LARGE induces *hyper*glycosylation of α-dystroglycan in both wild type and in cells from dystroglycanopathy patients, irrespective of their primary gene defect, restoring functional glycosylation. Viral delivery of LARGE to skeletal muscle in animal models of dystroglycanopathy has identical effects *in vivo*, suggesting that the restoration of functional glycosylation could have therapeutic applications in these disorders. Pharmacological strategies to upregulate Large expression are also being explored.

**Methodology/Principal Findings:**

In order to asses the safety and efficacy of long term LARGE over-expression *in vivo*, we have generated four mouse lines expressing a human LARGE transgene. On observation, LARGE transgenic mice were indistinguishable from the wild type littermates. Tissue analysis from young mice of all four lines showed a variable pattern of transgene expression: highest in skeletal and cardiac muscles, and lower in brain, kidney and liver. Transgene expression in striated muscles correlated with α-dystroglycan hyperglycosylation, as determined by immunoreactivity to antibody IIH6 and increased laminin binding on an overlay assay. Other components of the dystroglycan complex and extracellular matrix ligands were normally expressed, and general muscle histology was indistinguishable from wild type controls. Further detailed muscle physiological analysis demonstrated a loss of force in response to eccentric exercise in the older, but not in the younger mice, suggesting this deficit developed over time. However this remained a subclinical feature as no pathology was observed in older mice in any muscles including the diaphragm, which is sensitive to mechanical load-induced damage.

**Conclusions/Significance:**

This work shows that potential therapies in the dystroglycanopathies based on LARGE upregulation and α-dystroglycan hyperglycosylation in muscle should be safe.

## Introduction

The muscular dystrophies (MDs) are a clinically and genetically heterogeneous group of conditions characterised by progressive muscle degeneration [1]. Defects in a wide variety of proteins with diverse functions in skeletal muscle are known to cause these disorders [1]. In the last few years the most common group of MDs after Duchenne muscular dystrophy has been shown to share the novel pathological feature of a *hypo*glycosylated form of α-dystroglycan (α-DG) [2], [3]. To date, mutations in seven genes encoding putative or demonstrated glycosyltransferases have been found to underlie these disorders, collectively known as secondary dystroglycanopathies: *Protein-O-mannosyl transferase1* (*POMT1*; OMIM 607423) [4], *Protein-O-mannosyl transferase 2* (*POMT2*; OMIM 607439) [5], *Protein-O-mannose 1,2-Nacetylglucosaminyltransferase1* (*POMGnT1*; OMIM 606822) [6], *Fukutin* (OMIM 607440) [7], *Fukutin-related protein* (*FKRP*; OMIM 606596) [8], [9], *LARGE* (OMIM 603590) [10] and Dol-P-Man synthase subunit 3 (DPM3, OMIM 605951) [11]. The clinical spectrum of dystroglycanopathies is uncommonly wide [3]. At the severe end of the spectrum are conditions characterised by forms of congenital muscular dystrophy (CMD) with severe structural brain and variable eye defects: Walker-Warburg-Syndrome (WWS [OMIM 236670]), Muscle-Eye-Brain disease (MEB [OMIM 253280]), Fukuyama CMD (FCMD [OMIM 253800]) and MDC1D [OMIM 608840]) [3]. Less severe phenotypes include one form of congenital muscular dystrophy, MDC1C [OMIM 606612] [9] and four forms of Limb girdle muscular dystrophy: LGMD 2I [OMIM 607155] [8], LGMD 2K [OMIM 609308] [12], LGMD 2M [OMIM 611588] [13] and the variant due to DPM3 deficiency [11]. Brain involvement is either mild (LGMD2K) or absent (LGMD2I and LGMD2M) in patients with the limb girdle phenotypes. Although each variant was originally associated with mutations in a particular gene, it is now clear that each of these genes can give rise to a similar broad phenotypic spectrum and that the severity of the condition is more dependent on the degree of perturbation of α-DG glycosylation than the gene primarily mutated [14]. No effective therapy for any of these progressive conditions exists [15].

Dystroglycan was originally identified as a component of the dystrophin associated glycoprotein complex (DGC) present at the sarcolemma of skeletal muscle [16], though it is now clear that it has a much wider tissue distribution [17]. The *DAG1* gene encodes a precursor protein that is post-translationally cleaved into α and βsubunits [18]. β-Dystroglycan (β-DG) is a 43kDa transmembrane protein whereas α-DG is an extracellular peripheral membrane protein non-covalently bound to the β subunit [16]. The full function of the DGC is not completely known although it forms a structural link between the extracellular matrix and the actin associated cytoskeleton [19]. The mature α-DG protein has globular N- and C-terminal domains separated by a central mucin rich domain [16]. The N-terminal domain (aa 29 to aa 312) appears to be cleaved by a convertase-like activity [20]. The predicted molecular mass of α-DG is 74 kDa but its apparent molecular weight ranges from 120 kDa (brain) to 156 kDa (skeletal muscle), due to tissue specific and developmentally regulated patterns of *O*-glycosylation within the mucin rich domain [16]. Structural information regarding the sugars decorating α-DG is limited [21]. However one structure that has received attention is the rare *O*-mannosyl linked oligosaccharide Neu5Ac (α2-3)Gal(β1-4)GlcNAc(β1-2)Man-Ser/Thr [22]. A complex of POMT1 and POMT2 is responsible for initiating its synthesis which is then extended by the action of POMGnT1 [23]. It was originally suggested that this mannosylated structure constituted a laminin receptor, though more recent studies have showed that enzymatic degradation of the terminal Neu5Ac actually results in increased laminin binding, suggesting other unknown structures also mediate this process [24].

Recently, using mass spectrometry and nuclear magnetic resonance (NMR) –based structural analyses, the group of Kevin Campbell identified a phosphorylated O-mannosyl glycan on recombinant α-DG, which was required for laminin binding [25]. This phosphorylation occurs on the O-linked mannose of α-DG. Further work from the Lance Wells' laboratory demonstrated that α-DG is mannosylated at 9 residues, while GalNAcylation occurs at 14 sites [26].

LARGE is a putative glycosyltransferase mutated in the myodystrophy mouse (LARGE-myd) [27] and in patients affected by MDC1D [10], one of the dystroglycanopathy variants associated with skeletal muscle and structural brain involvement. Sequence analysis predicts LARGE to contain two catalytic domains [28], [29]. The first domain is related to bacterial α-glycosyltransferases, while the second is most closely related to human β-1,3-*N*acetylglucosaminyltransferase, required for synthesis of the poly-*N*-acetyllactosamine backbone (Galβ1-4GlcNAcβ1-3)n found on *N*- and *O*-glycans [28]. Although neither of these structures is present on α-DG [21], there is strong evidence that LARGE plays a pivotal role in the functional glycosylation of α-DG. Firstly, the N-terminal domain of α-DG interacts directly with LARGE and this association is a requirement for physiological glycosylation [20]. Secondly, the forced overexpression of LARGE in mouse skeletal muscle, as well as cultured human and mouse cell lines, results in increased expression of functionally glycosylated α-DG (*hyper*glycosylation) and a corresponding increase in its binding capacity for laminin and other ligands [30], [31]. Moreover, the overexpression of LARGE generates highly glycosylated α-DG in cell lines derived from patients with a dystroglycanopathy, irrespective of the underlying gene defect [30]. While the precise nature of the LARGE induced glycosylation remains undetermined, it has been suggested that LARGE requires mannosylated α-DG to exert its action [25]. Furthermore LARGE gene transfer experiments achieved α-DG hyperglycosylation in animal models of fukutin and PomGnt1 related muscular dystrophies [32], thus LARGE overexpression can presumably activate alternative pathways resulting in functional α-DG glycosylation in these models. None of the other enzymes responsible for dystroglycanopathies has a similar effect; however we have previously demonstrated that the overexpression of the LARGE paralog GYLTL1B (or LARGE2) is equally capable of hyperglycosylating α-DG in cultured cells [29], [31], [33]; mutations in this gene have not yet been associated with a human pathology.

The presence of alternative pathways of α-DG glycosylation opens new avenues for the development of therapies in dystroglycanopathies [15], [30], [34], [35]. Overexpression of LARGE (or LARGE2) by means of genetic or pharmacological intervention could restore ligand binding and improve muscle strength in patients affected by dystroglycanopathies. However, prior to a therapeutic strategy based on the over-expression of LARGE being considered, an assessment of the safety of its long term over-expression and its efficacy with respect to the hyperglycosylation of α-DG needs to be established *in vivo*. To this end we report here the generation of four lines of LARGE overexpressing transgenic mice. We have characterised the effect of transgene expression on α-DG glycosylation in skeletal and cardiac muscle and brain, tissues which are affected in dystroglycanopathy patients, as well as other tissues not involved in these disorders. We show that the overexpression of LARGE results in a robust hyperglycosylation of α-DG in skeletal and cardiac muscle without any observable deleterious morphological effect. Detailed analysis of the contractile properties of tibialis anterior muscles however showed a loss of force in response to eccentric exercise in older mice. This was not accompanied by any morphological changes suggesting a mild subclinical defect. α-DG was not hyperglycosylated in brain despite low levels of expression of the transgene, which suggests that higher levels of LARGE are necessary to achieve hyperglycosylation in a tissue, in which high levels of endogenous Large are present.

## Results

### The generation of LARGE overexpressing transgenic mice

**Figure 1:**
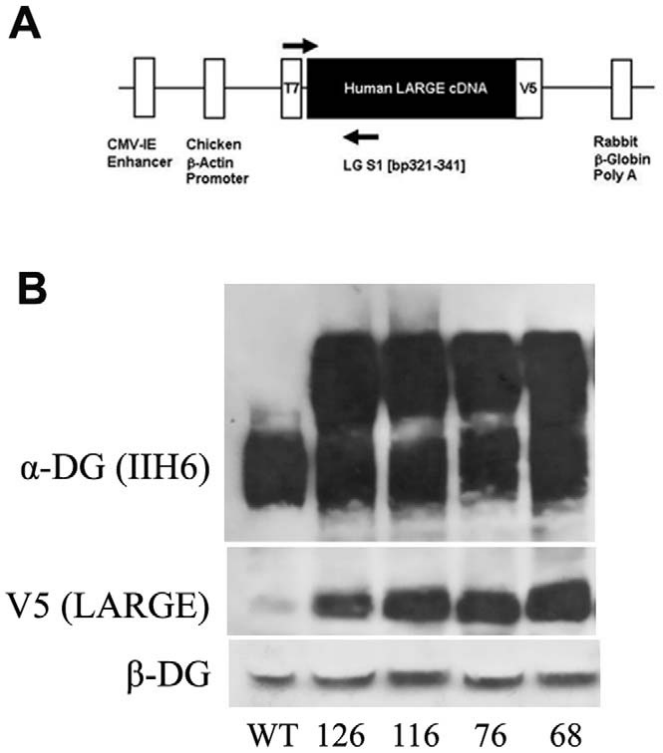
Generation of LARGE transgenic mouse. **A**. A schematic representation of the LARGE expression cassette. The full-length V5 tagged LARGE cDNA was subcloned into the pCAGGS expression vector. This vector consists of a synthetic CMVenhancer/and chicken β-actin promoter sequence located upstream of the transgene and the rabbit β-globin poly(A) sequence located downstream. Arrows represent position and orientation of PCR primers used to screen ear biopsies. **B**. Western blotting analysis of protein lysates from wild type and muscle tissues (quadriceps) from all the four LARGE transgenic lines (68,76,116 and 126) using antibodies to α-DG IIH6, β-DG, and V5.

In order to generate transgenic mice we cloned human LARGE into the pCAGGS expression vector which contains a human cytomegalovirus enhancer situated upstream of the chicken β-actin promoter and a rabbit β-globin 3′ flanking sequence including a polyadenylation signal [36], [37]. Since antibodies to human LARGE are not routinely available, the LARGE cDNA derived from total human brain RNA was initially cloned into the pcDNA 3.1/V5-His expression vector. This directs the synthesis of a fusion protein with the V5 epitope at the C terminal end. The DNA sequence coding for this fusion product was subsequently subcloned into pCAGGS (Figure 1). The transgene expression vector harbouring the LARGE/V5 fusion sequence was digested to release a 4kb cassette for micro injection. Founders were identified by PCR from ear biopsies and were used to establish independent transgenic lines by breeding to wild-type F1 hybrid (C57BL10×CBA/Ca) mice. Successful transmission of the transgene was identified by a further round of PCR screening. Expression of the transgene was confirmed by western blot analysis and immunocytochemistry using a V5 antibody. In total we identified seven transgenic founders, four of which transmitted and expressed the transgene (lines 68, 76, 116, 126), one died shortly after birth and the remaining two failed to transmit. The four transgenic lines showed similar expression of the transgene and α-DG hyperglycosylation (Fig 1B). Immunohistochemical analysis was also comparable between all four expressing transgenic lines.

### LARGE transgenic mice are phenotypically indistinguishable from their non transgenic litter mates

LARGE transgenic mice from each of the transmitting lines showed no detectable differences in mortality, behaviour, mobility and rate of weight gain compared to their non-transgenic littermates under standard husbandry conditions. Breeding performance was typical of the C57BL10×CBA/Ca hybrid with normal litter sizes and survival to weaning.

### LARGE transgene expression in skeletal muscle resulted in α-DG hyperglycosylation but had no effect on muscle morphology

**Figure 2:**
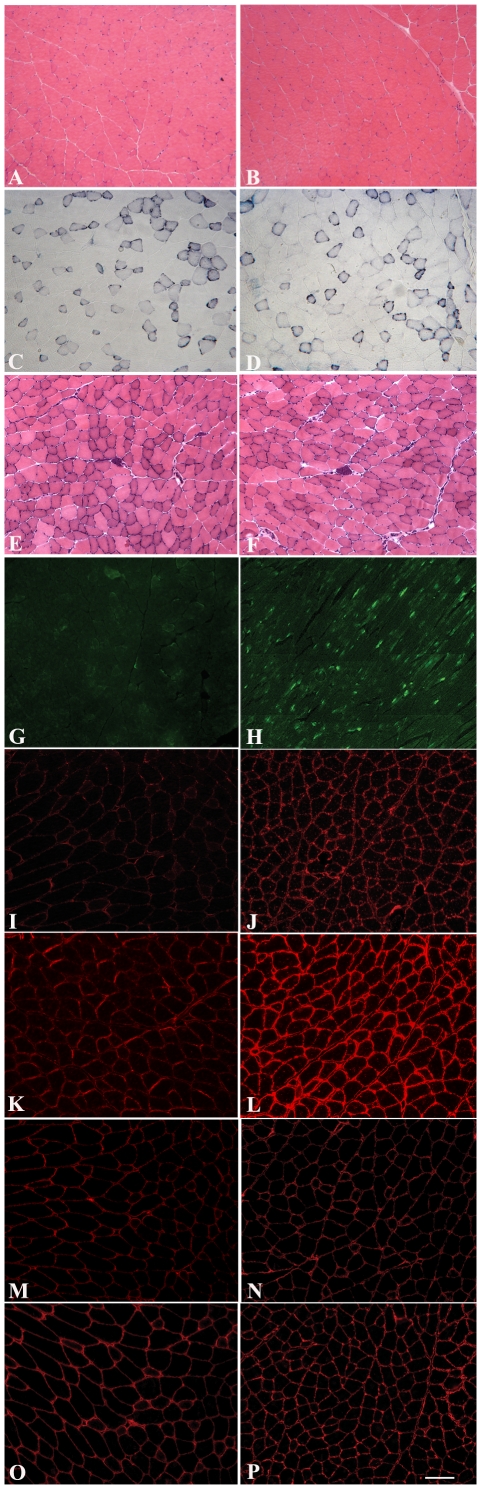
Immunocytochemistry of LARGE transgenic mouse skeletal muscle. Serial transverse frozen sections (except longitudinal section H) of *quadriceps* skeletal muscle from normal (A,C,I,M and O) and transgenic LARGE mouse line 68 (B,D,G,H,J,N and P) aged 2 months. E and K from control and F and L from transgenic LARGE mouse (line 68) aged 8 months. A,B,E and F: Haematoxylin and Eosin staining. C and D: NADH staining. Immunolabeling of V5 tagged LARGE in transverse (G) and longitudinal (H) section. V5 immunolabelling was only seen in few fibres in transverse section whereas in the longitudinal section all fibres appeared to be labelled. I–L immunolabelling of α-DG (IIH6). All the fibres in the LARGE transgenic muscle (J and L) showed a stronger staining compared to the control littermates (Iand K). No difference was seen in the level of β-DG (M and N) and laminin α2 (O and P) in LARGE transgenic muscle relative to the control. (Scale bar 50 µm).

**Figure 3:**
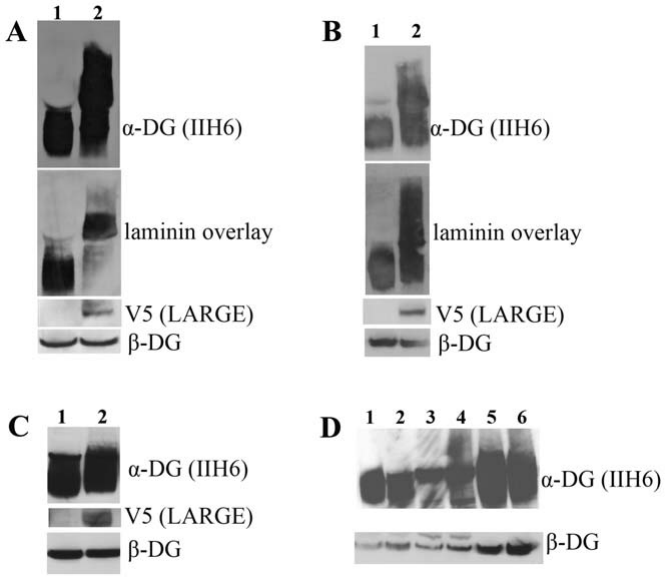
Western blot and laminin assay analysis of LARGE transgenic mouse tissues. Western blot analysis of protein lysates from wild type and LARGE transgenic (line 68) tissues using antibodies to α-DG IIH6, β-DG, and V5. **A**. Wild type and LARGE transgenic *quadriceps* muscle. When the membrane was exposed for a short period using antibody IIH6 a clear band of higher molecular weight was detected in the samples from LARGE transgenic muscle compared to controls, while a longer exposure resulted in a continuous smear. Laminin-1 overlay showed that hyperglycosylated α-DG has increased laminin binding. β-DG expression was unaltered in transgenic mice. **B**. Wild type and LARGE transgenic mouse cardiac muscle. Laminin-1 overlay showed that hyperglycosylated α-DG bound laminin with a similar capacity to normally glycosylated dystroglycan. **C**. Wild type and and LARGE transgenic total brain lysates. No hyperglycosylated α-DG was seen using IIH6 in the LARGE transgenic brain samples even though the LARGE transgene (V5) was clearly expressed. **D**. Western blot analysis of protein lysates from control (1,3 and 5) and LARGE transgenic (2,4,and 6) mice derived from: small intestine (1,2), liver (3,4) and kidney (5,6). No hyperglycosylated α-DG was seen in any of these tissues. Expression of V5 (LARGE) was only detectable in heavily overexposed blots (data not shown). α-DG expression was unaltered in all tissue samples.

Histological analysis of each of the lines using haematoxylin and eosin (H&E) staining showed no overall changes in muscle morphology in either the *quadriceps*, *soleus, tibialis anterior* or *extensor digitorum longus* of transgenic compared to aged matched non transgenic litter mates (aged 2–4 months). Centrally located nuclei, tissue fibrosis, necrosis and fatty infiltration, typical features of dystrophy, were never observed (Figure 2A–B). Skeletal muscle from transgenic and non transgenic mice was similar in terms of fibre type composition as judged by NADH histochemical staining (Figure 2C–D). LARGE transgene expression, detected using an antibody to the V5 epitope, showed staining in only a proportion of fibres in transverse section (Figure 2G). The analysis of longitudinal sections confirmed that only discrete areas of each fibre were V5 positive (Figure 2H) and this accounted for the apparent lack of staining of several muscle fibres in transverse sections. Immunostaining of transgenic skeletal muscle sections with antibody IIH6 (kind gift from Dr KP Campbell), which recognises a laminin binding site on α-DG [37], [38] showed a marked increase in immunolabelling in all muscle fibres of each of the transgenic lines (Figure 2I–J). No difference between muscle groups or between slow and fast fibres was observed (data not shown). Immunostaining with antibodies to β-DG (Figure 2M–N) and laminin-α2 (Figure 2O–P) was identical to non transgenic litter mates. Western blotting of protein lysates from *quadriceps* muscle demonstrated an increase in IIH6 immunoreactivity, appearing as a broad smear on nitrocellulose membranes that encompassed both wild type α-DG glycosylation and significantly higher molecular weight products (Fig 3A). A laminin overlay assay showed a corresponding increase in its binding capacity relative to wild type dystroglycan (Figure 3A). The expression of β-DG on western blot was unaltered (Figure 3A). Limb muscle morphology was also studied in older transgenic mice (8 months) from three different lines; no difference from aged control littermates was found (Fig 2E–F). Immunostaining with IIH6 (Fig 2K–L) showed that hyperglycosylated α-DG was still expressed in the old LARGE transgenic mice. Real-time qPCR in these older transgenic mice showed persistent expression of the transgene at levels similar to those in young mice (data not shown). The morphology of neuromuscular junctions in one transgenic line was also investigated in detail since dystroglycan and its glycosylation play a central role in the development/maintenance of these structures [39]. Using fluorescently tagged α-bungarotoxin we visualised both the general organisation of the acetylcholine receptors (AChRs) and the area of the postsynaptic membrane in the diaphragm. These analyses failed to detect any gross differences between transgenic and non transgenic mice (data not shown). In addition, we compared the expression of two unique glycoforms of α-DG restricted to the NMJ that are recognised by the two cytotoxic T antigens (CT1 and CT2; antibodies kindly provided by Dr PT Martin) to see if they were altered in the presence of hyperglycosylation [40]. We could observe no differences between transgenic and non transgenic mice in the expression of these NMJ antigens (data not shown).

### LARGE transgene expression in skeletal muscle does not affect force generation but increases the susceptibility to contraction-induced injury

**Figure 4:**
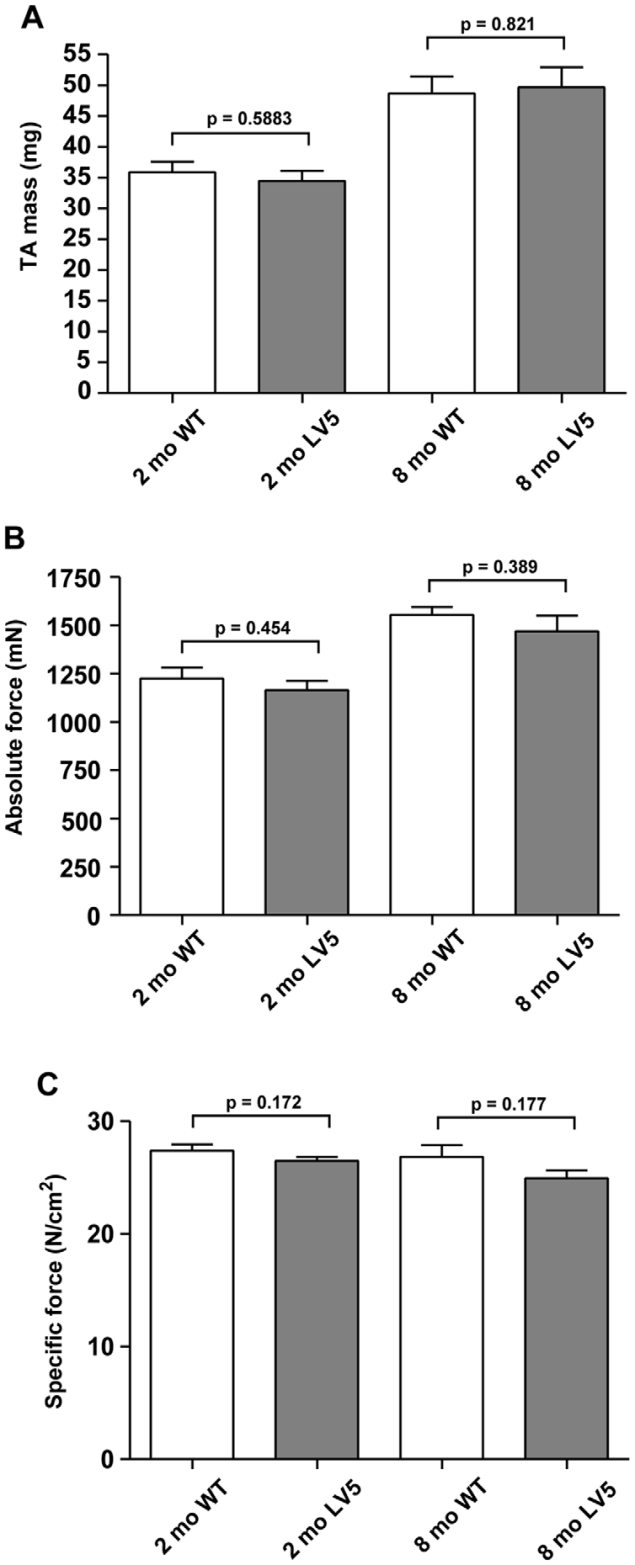
Isometric force contractions measurement in LARGE transgenic and the wild-type mouse muscle. Maximum isometric forces of tibialis anterior (TA) muscles from 2 and 8 month old LARGE transgenic mice (LV5, line 126) and their age-matched wild-type littermates (WT) were recorded *in vivo* and weighed following physiological assessments. (A) The weights of TA muscles from 2 month (2 mo) and 8 month (8 mo) old LARGE trangenics (LV5) were not significantly different from the TA muscles of the age-matched wild-type controls. (B and C) Both the maximum absolute forces (B) and the specific forces (C) of TA muscles from LARGE transgenics (LV5) at both 2 and 8 months of age were not significantly different from their age-matched wild-type controls. Number of animals examined: 2 months, LV5 n = 6; WT n = 4; 8 months, LV5 n = 4, WT n = 4. All values presented are mean and standard error of the mean (S.E.M).

**Figure 5:**
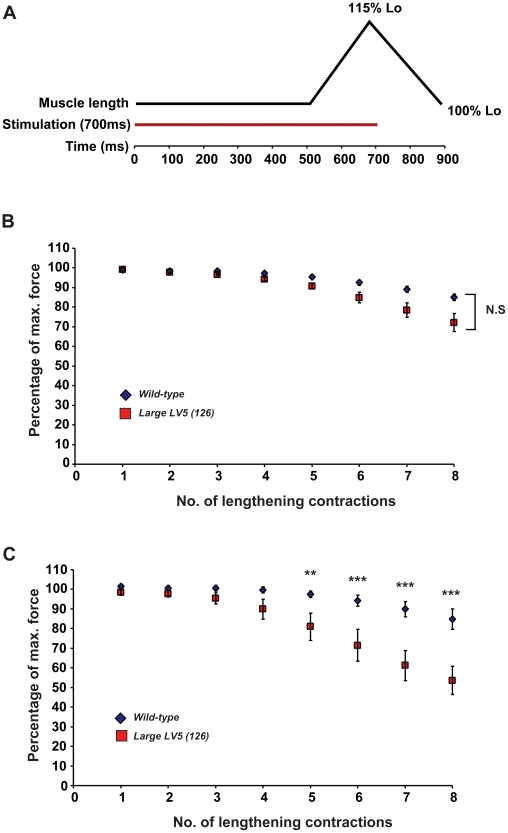
*In situ* Muscle Function and Lengthening Contraction. (A) Schematic representation of the lengthening contraction protocol, designed to assess the muscles resistance to contraction-induced injury. TA muscles from both LARGE transgenics (line 126) and wild-type controls were subjected to series of lengthening contractions and the effect on force generation was measured. (B) In two month old mice there was a significant effect of the number of eccentric contractions on force drop, however, there was no significant difference between LARGE transgenic mice (n = 6) and their non-transgenic littermates (n = 4). (C) In contrast in 8 month old mice the number of eccentric contractions had significant effects on the force drop (p = 0.04 and <0.001, respectively). There was no difference between LARGE transgenic mice (n = 4) and their negative littermates (n = 4) when the number of eccentric contractions was less than 5, but there were significant differences between LARGE transgenic and wild-type mice at 5, 6, 7 and 8 eccentric contractions (p = 0.01, 0.0007, <0.0001 and <0.0001 respectively). All values presented are mean and standard error of the mean (S.E.M).

We next examined whether overexpression of LARGE in transgenic mice would have an impact on skeletal muscle function. In order to achieve this, we measured *in vivo*, the isometric force contractions of tibialis anterior (TA) muscles from LARGE transgenic and the wild-type littermates at 2 and 8 months of age. At both ages we observed no significant differences in the weights, maximum absolute forces and specific forces of TA muscles from LARGE transgenics compared to wild-type controls (Fig. 4). We also tested the possibility that LARGE overexpression may alter the resistance of TA muscles to contraction-induced injury. Following the muscle force assessment, TA muscles were subjected to a series of lengthening contractions (15% of L_o_, Fig. 5A), which imposes additional stress on the sarcolemmal membrane of muscle fibres. The impact of these repeated lengthening contractions on force generation was measured over time. We observed no significant difference in resistance to contraction-induced injury in 2 month old LARGE transgenic mice compared to control mice (Fig. 5B) However, at 8 months of age, LARGE transgenic mice developed a significant susceptibility to contraction-induced injury, as demonstrated by a 30% greater decline in force generation compared to controls following 8 successive lengthening contractions (Fig. 5C). Even though there continued to be no weight or phenotypic differences between the transgenic and non-transgenic littermates prior to the assessment of muscle physiology at 8 months of age, we examined diaphragms from 9 month old transgenic mice as this muscle undergoes repeated eccentric exercise but did not observe any signs of pathology (data not shown).

### LARGE transgene expression in cardiac muscle was not uniform and correlated with a variable pattern of α-DG hyperglycosylation

**Figure 6:**
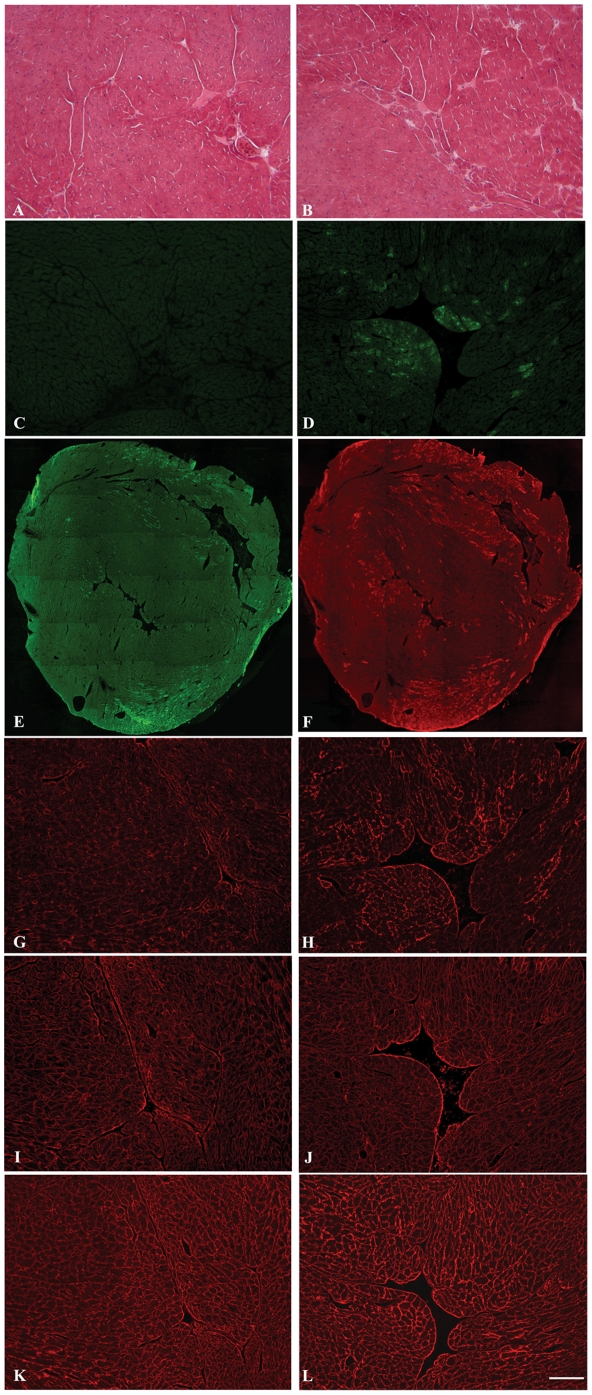
Immunocytochemistry of LARGE transgenic mouse cardiac muscle. Serial cryosections of control (A,C,G,I and K) and transgenic (line 68) (B,D–F, H, J and L) cardiac muscle (aged 2 months) stained with: haematoxylin and eosin (A–B), anti-V5 (C–E), α-DG (IIH6) (F–H), β-dystroglycan (I–J) and laminin α2 (K–L). E and F are images of whole heart sections of D and H. These images clearly show that the LARGE transgene is only expressed in a proportion of cardiomyocytes and that α-DG hyperglycosylation is confined to those cardiomyocytes positive for V5. No difference was seen in the level of β-DG (I and J) and laminin-α2 (K and L) immunoreactivity in LARGE transgenic cardiac muscle relative to control littermates. (Scale bar 50 µm).

Histological analysis using haematoxylin and eosin staining of sections of cardiac muscle from 2–4 month old transgenic mice from each the four lines showed no differences compared to aged matched non transgenic littermate controls (Figure 6A–B). Immunohistochemical staining of cardiac muscle sections with a V5 antibody showed a regional and patchy distribution of LARGE transgene expression (Figure 6D). Approximately 25% of cardiomyocytes had V5 expression and these were often but not invariably grouped with a transmural distribution (Figure 6E). There was a good correlation between cardiomyocytes showing α-DG hyperglycosylation and transgene expression (Figure 6E–F and 6D–H). Immunostaining with antibodies to β-DG (Figure 6I–J) and laminin-α2 (Figure 6K–L) showed no differences from non transgenic litter mates. Western blotting of cardiac muscle protein lysates from transgenic mice using the α-DG antibody IIH6 showed the presence of two distinct bands (Figure 3B). The lower band, which was equivalent to that seen in non transgenic litter mates, presumably represents the form of α-dystroglycan in cardiomyocytes not expressing the transgene, while the upper band likely represents hyperglycosylated α-DG produced by cardiomyocytes expressing the transgene. An overlay assay showed that both species of α-DG bound laminin-1 with equal efficiency (Figure 3B). The expression of β-DG was unaltered and V5 expression was only seen in the transgenic tissues (Figure 3D).

### LARGE transgene expression in brain was variable and did not induce α-DG hyperglycosylation

**Figure 7:**
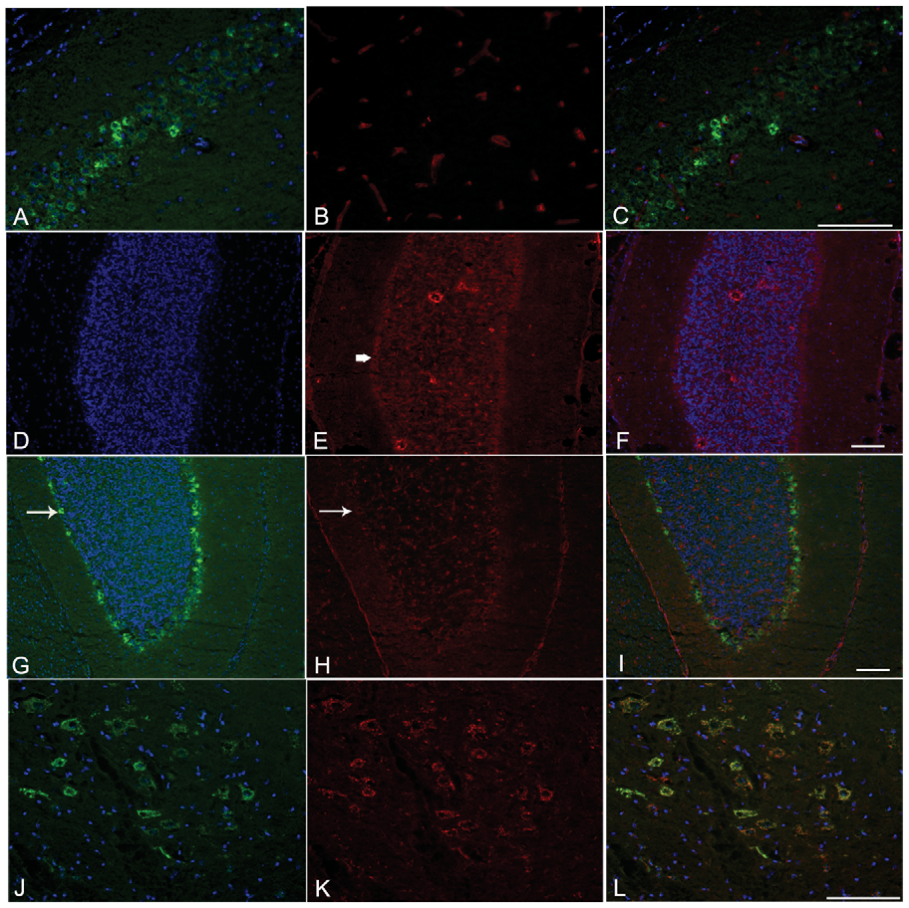
Immunocytochemistry of LARGE transgenic mouse brain. Cryosections of brains from LARGE transgenic (line 68) (A–C,G–L) and wild type littermates (D–F) at 2 months of age. Sections were stained using antibodies: V5 (A,D,G,J); α-DG (IIH6) (B,E and H); GM130, a Golgi marker, (K). Nuclei were stained with Hoechst 33258 (blue). C,F,I and L are merged images. Cells expressing different levels of V5 (LARGE) were seen in the hippocampal pyramidal layer (A) while IIH6 α-DG stained only vessels (B). In the cerebellum, immunolabelling of V5 was detected in Purkinje cells (G, white arrow). In these cells IIH6 α-DG was seen around the cell soma (H, arrow), in a similar level to wild type Purkinje cells (E, arrow). Colocalisation of V5 and GM130 in neuronal cells of the cerebral cortex (L) shows that the transgene was localised to the Golgi.

Immunohistochemical analysis of transgenic mouse brains from all four lines showed regional expression of the LARGE transgene as detected by V5 staining, including the hippocampus (Figure 7A–C), cerebellum (Figure 7G–I) and cortex (Figure 7J–L), though even within these areas expression was variable. Transgene expression, as judged by double staining with the neuronal markers NeuN and Ctip2, appeared to be largely restricted to neurons (data not shown). In the cerebellum, expression was further largely restricted to Purkinje cells (Figure 7G). We could not detect a clear increase in the levels of IIH6 immunoreactivity in cells expressing the transgene (Figure 7G–I) compared to control (Figure 7D–F). A double staining using an antibody against a marker for the Golgi apparatus (GM130) and V5 showed a clear co-localisation in neuronal cells, demonstrating that the LARGE transgene was properly localised in these cells (Figure 7J–L). Western blotting of transgenic brain lysates using the antibody IIH6 failed to identify α-DG hyperglycosylation in brain tissue (Figure 3C).

### The LARGE transgene was expressed at low levels in kidney, liver and small intestine but did not induce α-DG hyperglycosylation

We also investigated LARGE transgene expression in tissues not implicated in the patho-physiology of the dystroglycanopathies. These were kidney, liver and smooth muscle (small intestine). Western blot analysis using the V5 antibody could only detect very low levels of transgene expression on overexposed gels (data not shown); α-DG was not hyperglycosylated in any of these tissues in any of the transgenic lines (Figure 3D).

### Real time qPCR analysis of LARGE transgenic tissues

**Figure 8:**
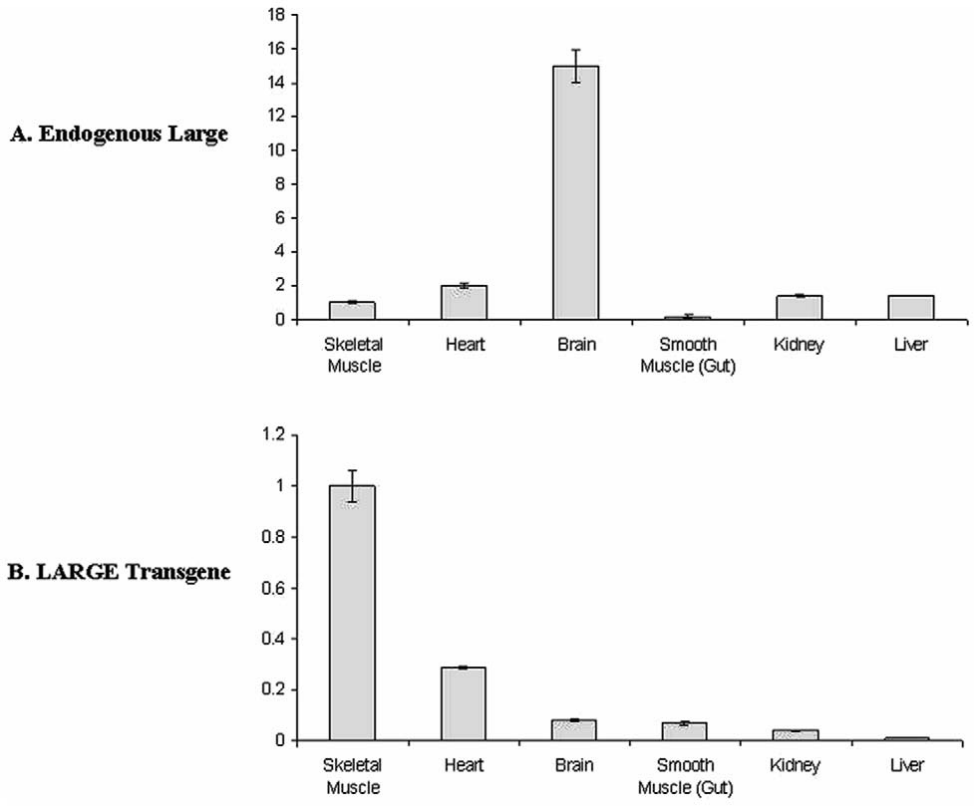
Tissue Expression of endogenous Large and transgenic LARGE. Real time qPCR analysis of endogenous Large and LARGE transgene expression is various tissues from transgenic line 115. All levels are relative to those seen in skeletal muscle ( = 1) and have been normalised using 18sRNA levels. No differences in levels of endogenous Large were seen between control littermates and transgenic mice. Error bars are standard errors based on 3 separate experiments.

In order to obtain further information on the levels of LARGE involved in inducing α-DG hyperglycosylation we used real time qPCR and TaqMan gene expression assays to quantify both the levels of endogenous Large and LARGE transgene expression. In wild type mice the expression of endogenous Large was found to be on average 15–20 fold higher in the brain and 2 fold higher in the heart relative to skeletal muscle (*quadriceps*) (Figure 8A). Skeletal muscle from transgenic mice had identical patterns of endogenous Large expression and therefore the presence of the LARGE transgene appeared not to affect levels of endogenous Large. Expression of the LARGE transgene in skeletal muscle was on average 10–15 fold that of brain and around 4 fold higher than heart (Figure 8B). Transgene expression in smooth muscle (small intestine), kidney and liver was at levels lower than brain. Probes to dystroglycan showed no differences in levels of expression of this gene (data not shown).

### LARGE hyperglycosylation does not affect the expression of other genes involved in α-DG glycosylation

Finally, we used real time PCR in transgenic *quadriceps* muscle of two month old mice (line 68) to investigate if LARGE transgene expression altered the expression of other known genes involved in dystroglycan glycosylation. These were *Pomt1*, *Pomt2*, *Pomgnt1*, *Fukutin*, *Fkrp* and the *Large* paralog *Large2* (*Gyltl1b*). No significant difference in the levels of expression of any of these genes was seen between aged matched transgenic and non transgenic littermates (data not shown).

## Discussion

Patients and animal models affected by dystroglycanopathies have a deficiency in functionally glycosylated α-DG [2], [34]. While the precise glycosylation pattern of α-DG is currently not known [21], several lines of evidence suggest that it is heterogeneous. α-DG is one of the few mammalian proteins known to contain *O*-mannosylated glycans [41] and now the sites which undergo this modification have been clearly mapped [26]. Three of the protein defects responsible for the dystroglycanopathies clearly participate in the mannosylation process. POMT1and POMT2 form a complex that confers full *O*-mannosyltransferase activity and POMGnT1 catalyzes the transfer of *N*-acetylglucosamine to *O*-mannosyl groups [23]. Regarding DPM3 deficiency, reduced dolichol-phosphate-mannose (Dol-P-Man) synthase activity has been associated with reduced O-mannosylation of α-DG [11].

The functions of the remaining 3 genes (*Fukutin*, *FKRP* and *LARGE*) remain elusive. The LARGE protein is unusual in that it is predicted to contain two putative catalytic domains [28]. Mutational analysis suggests that both domains are required for its biochemical function [31]. LARGE is ubiquitously expressed and is the only member of this group of proteins (together with LARGE2) whose overexpression induces hyperglycosylation of α-DG as judged by increased immunoreactivity to antibodies IIH6 and VIA41 both of which are known to recognise carbohydrate epitopes [29], [31]. Increased IIH6 immunoreactivity is accompanied by an increase in laminin binding capacity, consistent with the IIH6 epitope constituting a functional laminin binding glycan [42]. Forced expression of LARGE is also capable of inducing the synthesis of the IIH6 antigen in primary cell cultures derived from patients with dystroglycanopathies [30]. More recently, acute intramuscular adenoviral gene transfer of *LARGE* in fukutin and POMGnT1 deficient mice has provided further proof that *in vivo* forced overexpression restores α-DG glycosylation and ligand binding [32]. Although originally it was suggested that LARGE could exert its action via the modification of *N*-glycans [43] rather than on *O*-mannosyl residues, recent observations indicate that this protein is involved in the phosporylation of mannosyl residues on α-DG [25]. Overall these data suggest that LARGE overexpression may be of therapeutic benefit to patients affected by a dystroglycanopathy, irrespective of the primary gene defect. Mutations in *LARGE* are exceptionally rare, with only a handful of patients with proven mutations being identified since the original description of MDC1D in 2003 [10]. This has led to the suggestion that the administration of pharmacologically active small molecules, capable of upregulating LARGE, or LARGE2 which however is essentially not expressed in muscle, or both, could be an interesting therapeutic strategy for a broad range of dystroglycanopathy patients [30], [31], [34].

In order to explore the efficacy and long term safety of LARGE over-expression *in vivo*, we generated 4 independent LARGE overexpressing transgenic mouse lines. As LARGE has previously been reported to be expressed in a wide range of adult tissues, with high levels in brain, heart, skeletal muscle, we wanted to demonstrate if its generalised overexpression was capable of inducing α-DG hyperglycosylation in these tissues and whether this was associated with any detrimental effects. The LARGE transgene was therefore put under control of a modified β-actin promoter, previously shown to drive robust ubiquitous expression in all tissues [44]–[46]. Interestingly whilst the expression of LARGE was noted in several tissues, the hyperglycosylation of α-DG was only observed in skeletal and cardiac muscles. In skeletal muscle all fibres expressed the LARGE transgene. Since LARGE is localised to a discrete subcellular region, most likely the Golgi apparatus [31], expression in transverse sections was patchy. However, longitudinal sections confirmed transgene expression in virtually all fibres and increased IIH6 immunoreactivity also in all fibres. As hyperglycosylation of α-DG could have affected its interaction with its extracellular matrix ligands we studied the skeletal muscle histology of young and aged mice (8 months). Histologically the young and old LARGE transgenic muscles were indistinguishable from littermate controls, including the diaphragm, a muscle particularly prone to pathological changes because of the very frequent mechanical load. Immunohistochemical studies of laminin α2 and β-DG were also identical to muscle from control littermates. As abnormal dystroglycan glycosylation has been shown to affect neuromuscular junctions (NMJ) [47], [48] we hypothesised that hyperglycosylation of α-DG may affect the expression of two unique NMJ glycoforms of α-DG recognised by the two cytotoxic T antigens (CT1 and CT2). However we could demonstrate no morphological abnormality of NMJs and the expression of CT1 and CT2 was unchanged in transgenic mice.

Detailed muscle physiological studies identified loss of force in response to eccentric exercise in the older mice. As this physiological deficit was absent in the younger mice, this suggests that it developed over time. The reason for this deficit is not clear. However basal lamina deposition and maintenance is a complex phenomenon, following the initial deposition of laminin and its transition from a monomeric to a polymerized state following interaction with cellular receptors such as dystroglycan and alpha7beta1_D_ integrin [49]. We can therefore hypothesise that the hyperglycosylation of α-DG alters some aspects of basement membrane turnover. Interestingly, a defect of glycosylated dystroglycan as present in the Large(myd) mice is also associated with reduced sarcolemmal integrity and damage induced by eccentric exercise, further indicating the role of the dystroglycan-linked basal lamina to the maintenance of sarcolemmal integrity and protection of muscles from damage [50]. The lack of obvious muscle pathology in the limb muscles and the diaphragm of old mice nevertheless suggests that this is a subtle, and subclinical defect.

LARGE transgene expression and α-DG hyperglycosylation was also detected in cardiac muscle in all transgenic lines; however in this organ there was a patchy expression of the transgene. We do not know why only a subset of cardiomyocytes expressed the transgene. To our knowledge this is a unique observation using the pCAGGs vector, which has been used extensively in the generation of transgenic mouse lines. Increased IIH6 immunoreactivity was restricted to regions of transgene expression. Histologically, there was no difference between the transgenic hearts compared to the non transgenic littermates.

When the pattern of LARGE transgene expression in the brain was studied, we demonstrated that its expression was restricted to a discrete number of cell types, including the hippocampus, cerebellum and cortex and associated primarily with neuronal cells. Unlike skeletal and cardiac muscles, however, transgene expression was not apparently associated with significant α-DG hyperglycosylation in any of the neurons. Western blotting analysis of total brain lysates confirmed what was seen at the cellular level namely that there was no α-DG hyperglycosylation.

The failure to achieve α-DG hyperglycosylation in transgenic mouse brains may be due to insufficient transgene expression in this tissue, although the promoter used has been previously shown to be ubiquitously expressed. However we were able to demonstrate that the brain has the highest levels of endogenous Large expression; we therefore hypothesise that the amount of LARGE transgene expression required to bring about a fold increase in overall protein levels is much greater in brain than in skeletal and cardiac muscles, which have considerably lower levels of endogenous Large expression. While this suggests that it might be more difficult to achieve hyperglycosylation in this tissue, it should be considered that the most severe structural brain defects seen in patients are developmental in origin and would not be helped by postnatal restoration of α-DG function [51].

The lack of any perturbation in the expression profiles of glycosyltransferases involved in DG glycosylation strengthens the argument that LARGE induced hyperglycosylation recruits a different set of enzymes and functions by activating an alternative enzymatic pathway. Our results further suggest that a strategy aimed at inducing hyperglycosylation of α-DG has no obvious detrimental effects on muscle histology, at least in mice. While we found a physiological impairment of old transgenic mice, this was not accompanied by any pathological changes. Although this suggests that the effect of hyperglycosylation strategies might need to be evaluated carefully, it is important to acknowledge both that the deficit was only subclinical and also that the transgenic overexpression of LARGE in a normal muscle differs radically from the situation in a muscle depleted of glycosylated α-DG epitopes, such as in the dystroglycanopathies, where the absence of a proper α-DG-laminin interaction plays a major pathogenic role. It will nevertheless be important to demonstrate safety and efficacy of this strategy on the muscle physiology phenotype of models deficient in enzymes involved in the glycosylation of α-DG. These results are also encouraging in view of the recent findings indicating that only modest restoration of IIH6 and laminin binding might be associated with a beneficial effect in muscle of a dystroglycanopathy model [32]. As the majority of patients affected by dystroglycanopathies, especially LGMD2I, are relatively mild variants with no evidence of brain involvement, a modest increase in α-DG hyperglycosylation such as could be achieved with pharmacological intervention, might be expected to produce a significant benefit in the function of skeletal and cardiac muscle, primary targets for the condition [52], [53].

## Materials and Methods

### Ethics Statement

All animal experiments were conducted under a Project Licence (PPL 70/6797) as mandated by the Animals (Scientific Procedures) Act (1986). The study conformed to the guidelines published by the Home Office in the UK and the local guidelines at Imperial College London.

### Generation of transgene cassette

Human LARGE cDNA derived from total brain RNA was cloned into the pcDNA 3.1/V5-His TOPO expression vector as reported previously [31]. This directs the synthesis of a fusion protein with the V5 epitope at the C terminal end. The entire LARGE/V5 DNA sequence was subsequently amplified by PCR using primers to the T7 and BGH reverse priming sites and blunt ligated into a *Bal*I linearised pCAGGS vector (nt1865). The transgene expression vector was then digested with *Hind*III and *Sal*I to release a 4kb cassette for micro injection. After purification the transgene was injected into the pronuclei of single cell fertilised eggs from C57BL10×CBA/Ca hybrid mice which were then placed in pseudo pregnant CD-1 foster mothers. Three weeks after birth DNA was extracted from small ear biopsies and integration of the transgene cassette was identified by PCR using a primer located within the LARGE coding sequence at nt361(LGS; CTGCCCAATGCTAAGATG) and one in the flanking portion of the cassette (T7 primer). Founder transgenics were then bred with C57BL10×CBA/Ca hybrid mice to establish independent transgenic lines for subsequent analysis.

### Immunocytochemistry and histology

Samples for histology and immunocytochemistry were collected from transgenic and non transgenic littermates following cervical dislocation and frozen on cork blocks with Cryoembed in isopentane cooled in liquid nitrogen before storage at −80°C. Cryostat sections (8 µm) were air-dried for 30 min at room temperature. Where mouse monoclonal antibodies were used the sections were pretreated with the M.O.M.™ Kit (Vector Labs) according the manufacturer's instructions, to reduce background or non-specific staining of mouse tissues. Sections were incubated with an anti-goat V5 FITC conjugated antibody (Bethyl laboratotries, USA) for 1 hour. For double staining, the sections were incubated with the following antibodies: α-DG IIH6 (a kind gift from KP Campbell), NeuN (Millipore,UK, MAB377), Ctip2 (Abcam, UK), GM130 (BD Biosciences, USA), laminin α-2 (Alexis Biochemicals, Enzo Life Sciences Ltd (UK)), β-DG (Vector Labs, UK). After washing, the sections were incubated with an appropriate biotinylated secondary antibody for 30min followed by incubation with streptavidin conjugated to Alexa 594 (Molecular Probes, Invitrogen,USA) for 20 minutes. All the incubations were at room temperature. Cell nuclei were stained with Hoechst 33258 (Invitrogen, USA). All dilutions and washings were made in phosphate buffered saline. Sections were mounted in aqueous mountant and viewed with epifluorescence using a Leica Diaphot microscope and digitally captured using Metamorph (Universal Imaging Inc). Control sections were used to set the exposure time and scale settings for the digital capturing system. Haematoxylin and eosin (H&E) and nicotinamide adenine dinucleotide dehydrogenase-tetrazolium reductase (NADH-TR) stainings were carried out in order to assess cell morphology, inflammatory infiltrate and fibre type.

### Western blotting and laminin overlay assays

Cell proteins were extracted in sample buffer consisting of 75 mM Tris–HCl, 1% SDS, 2-mercaptoethanol, plus a cocktail of protease inhibitors (Roche). 30ug of soluble proteins were resolved using a NuPage Pre-cast gel (4–12% Bis–Tris; Invitrogen, USA) and then transferred electrophoretically to nitrocellulose membrane (Hybond-ECL, GE Healthcare, UK. Nitrocellulose strips were blocked in 3% BSA (IgG and protease-free, Jackson Laboratories, USA) in Tris-buffered saline buffer, and then probed with the primary antibodies: anti mouse α-DG IIH6 (Millipore UK,cat,05-593) anti-mouse β-DG (Vector Labs,UK), anti-mouse V5 (Invitrogen, USA) at room temperature for 1 hour. After washing they were incubated with the appropriate biotinylated secondary antibody: anti IgM (Dako,Denmark), anti mouse IgG (GE Healthcare, UK) followed by a HRP-streptavidin (Dako, Denmark). All the incubations were for 1 hour at room temperature. After washing, membranes were visualized using chemiluminescence (ECL+Plus,GE Healthcare, UK). For the laminin overlay assay, nitrocellulose membranes were blocked for 1 hour in laminin binding buffer (LBB: 10 mM triethanolamine, 140 mM NaCl, 1 mM MgCl2, 1 mM CaCl2, pH 7.6) containing 5% non-fat dry milk followed by incubation of mouse Engelbreth-Holm-Swarm laminin (Invitrogen,USA) overnight at 4°C in LBB. Membranes were washed and incubated with anti rabbit laminin (Sigma, USA) followed by HRP-anti rabbit IgG (Jackson ImmunoResearch, USA). Blots were visualized using chemiluminescence (ECL+Plus, GE Healthcare,UK).

### Immunocytochemistry for CT antigens

10 µm transverse sections of the Gastrocnemius were blocked with 3% BSA (Sigma) in PBS for 1h. The CT1 and CT2 antibodies (a kind gift from Paul T. Martin) were diluted in 3% BSA/PBS, and the slides incubated overnight at 4°C. The following day, slides were incubated for 1hour with a biotinylated anti-mouse IgM (Molecular Probes, Invitrogen,USA) followed by incubation with streptavidin Alexa 594 (Molecular Probes, Invitrogen, USA). NMJs were visualised using alpha-bungarotoxin conjugated with Alexa 488 (Molecular Probes, Invitrogen, USA) and the nuclei stained with Hoechst 33342 (Molecular Probes, Invitrogen, USA). Sections were mounted in aqueous mountant and viewed with epifluorescent using a Nikon Eclipse 1000, and digitally captured with Imaging Software Pro Plus. Appropriate controls were performed with secondary antibodies only.

### Neuromuscular Junction Morphology

Diaphragms from normal and transgenic mice were dissected, fixed in 4% paraformaldehyde and stained en bloc. They were first incubated in PBS plus Triton 0.5% for 10 min followed by incubation with of α-bungarotoxin Alexa 488 (Molecular Probes, Invitrogen, USA) for 10 minutes. Nuclei were stained with Hoechst 33342 and sections were then mounted in aqueous mountant. Images were captured and the area of muscle fibre covered by the NMJ measured using a Leica DMR epifluorescence microscope interfaced with Metamorph software. Only those neuromuscular junction images where the full area was visible were analysed.

### RT-PCR analysis

Tissues of interest were dissected out and homogenized with liquid nitrogen using a mortar and pestle and the lysate passed through a QiaShredder (Qiagen, USA). The RNA was isolated from the homogenized tissue using an RNeasy kit (Qiagen,USA) and eluted in 30 µl. About 1 µg of RNA was reverse transcribed with Superscript III for qRT-PCR kit (Invitrogen,USA). qRT-PCR was performed on a 7500 FAST Real-Time PCR system (Applied Biosystems,USA) using TaqMan Universal PCR Mastermix and TaqMan gene expression assays (Applied Biosystems, USA). Expression levels of transgenic LARGE and endogenous Large were normalised using 18sRNA levels. All reactions were performed in triplicate.

### In situ Muscle Function and Lengthening Contraction

Mice were anaesthetised with an intra-peritoneal injection of Hypnorm/Hypnovel/water (1∶1∶2 by vol.) at a dosage of 10 ml/kg (Hypnorm, VetPharma,UK and Hypnovel,Roche,UK). The mice were carefully monitored throughout the experiment and additional doses of anaesthetic were administered to ensure that there was no reflex response to toe pinch. Under deep anaesthesia the distal tendon of the tibialis anterior (TA) muscle was dissected from surrounding tissue and the tendon tied with 4.0 braided surgical silk. The sciatic nerve was exposed and all its branches cut except for the common peroneal nerve (CPN), which innervates the TA muscle. The mouse was placed on a thermopad (Harvard Apparatus) to maintain body temperature at 37°C. The foot was secured to a platform and the ankle and knee immobilized using stainless steel pins. The TA tendon was attached to the lever arm of a 305B dual-mode servomotor transducer (Aurora Scientific Inc., Ontario, Canada) via a custom made steel s-hook. TA muscle contractions were elicited by stimulating the distal part of CPN via bipolar platinum electrodes, using supramaximal square-wave pulses of 0.02 ms (701A stimulator; Aurora Scientific Inc). Data acquisition and control of the servomotors were conducted using a Lab-View based DMC program (Dynamic muscle control and Data Acquisition; Aurora Scientific, Inc.). Optimal muscle length (L_o_) was determined by incrementally stretching the muscle using micromanipulators until the maximum isometric twitch force was achieved. The peak twitch force (Pt), time to peak twitch force (TPT), half relaxation time of the twitch (HRT) and the specific twitch force (sPt, Pt normalised for cros sectional area) was recorded for each mouse. The maximum isometric tetanic force (P_o_) was determined from the plateau of the force-frequency relationship following a series of stimulations at 10, 30, 40, 50, 80, 100, 120, 150, and 180 Hz. A one minute rest period was allowed between each tetanic contraction. Muscle length was measured using digital calipers based on well defined anatomical landmarks near the knee and the ankle. The specific force (N/cm^2^) was calculated by dividing P_o_ by TA muscle cross-sectional area (CSA). Overall CSA was estimated using the following formula: muscle weight (g)/[L_o_ (cm)×1.06 (g/cm^3^).

After establishing the force-frequency relationship, the susceptibility of TA muscles to eccentric contraction-induced injury was assessed. This consisted of stimulating the muscle at 180 Hz (the frequency that usually resulted in P_o_) for 700 ms. After 500 ms of stimulation the muscle was lengthened by 15% of L_o_ at a velocity of 0.75 L_o_ s/1. At the end of stimulation the muscle was returned to L_o_ at a rate of −0.75 L_o_ s/1. The stimulation-stretch cycle was repeated every 3 min for a total of 10 cycles. The rest time between each cycle limited the potential for developing muscle fatigue. Maximum isometric force was measured after each eccentric contraction and expressed as a percentage of the initial maximum isometric force. At the end of the experiment the muscles were excised and weighed.

Statistics: Mixed effect models were employed to study the effect of genotype, number of eccentric contractions and their interaction on outcome variables, and first-order autoregressive structure was adopted to account for the correlation structure between repeated measures from the same mouse. These analyses were carried out using the MIXED procedure in SAS version 9.1 for PC (Copyright, SAS Institute Inc. Cary, NC, USA).
